# Strategic Key Performance Indicators for AI in Lead Optimization

**DOI:** 10.1002/cmdc.202501089

**Published:** 2026-03-22

**Authors:** Theodor Theis, Stefanie Flohr, Hayley Binch, Werngard Czechtizky, Ewa Chudyk, Markus Klein, Mireille Krier, Franz von Nussbaum

**Affiliations:** ^1^ Medicinal Chemistry Boehringer Ingelheim Pharma GmbH & Co KG Biberach an der Riß Germany; ^2^ Novartis Institute of Biomedical Research Novartis AG Basel Switzerland; ^3^ Roche Pharmaceutical Research and Early Development Roche Innovation Center Basel Basel Switzerland; ^4^ Medicinal Chemistry Research & Early Development, Respiratory & Immunology, BioPharmaceutical R&D AstraZeneca Mölndal Sweden; ^5^ Digital Life Science Chemistry Nuvisan ICB GmbH Berlin Germany; ^6^ Merck KGaA Darmstadt Germany; ^7^ Application Science OpenEye, Cadence Molecular Sciences Cologne Germany; ^8^ Digital Drug Discovery & Development Nuvisan ICB GmbH Berlin Germany

**Keywords:** artificial intelligence, computational chemistry, key performance indicators

## Abstract

With increasing cost and failure rates in the pharmaceutical R&D process not fundamentally improving over the last decade, pressure remains high to increase the probability of success to improve the effectiveness of pharmaceutical R&D. The broad introduction of AI into the R&D landscape over the last years holds the promise to lift pharmaceutical R&D out of its productivity problem, as preliminary analyses suggest that “AI‐native” companies may be outpacing traditional peers. However, harnessing this potential requires moving beyond measuring technical model performance (e.g., predictive accuracy) to measuring strategic impact. In this perspective, members of the EFMC^2^ community—focused on advancing the collaboration between computational and medicinal chemists—discuss the challenges of applying key performance indicators (KPIs) in the idiosyncratic environment of drug discovery. We argue that the shift from expert‐driven computer‐aided drug design (CADD) to semiautonomous AI necessitates a new framework of impact‐oriented KPIs. We provide recommendations for designing these strategic indicators to drive adoption, foster innovation, and objectively assess whether digital tools are delivering top‐line impact.

## Graphical abstract

As AI becomes an integral assistant in lead optimization, measuring its true value requires a shift from technical metrics to strategic key performance indicators (KPIs). This perspective from the EFMC^2^ initiative recommends specific, impact‐oriented KPIs to objectively assess how digital tools enhance decision‐making and impact top‐line results.

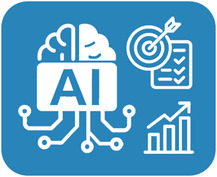

## 1 Introduction

The management of knowledge workers has been a central theme in modern organizational theory [1, 2], largely shaped by the pioneering work of Peter Drucker [3]. He argued that unlike industrial labor, knowledge work thrives on autonomy and innovation [4]—a perspective particularly relevant in drug discovery, where success depends on creativity and iterative problem‐solving. To effectively manage such complex work, organizations rely on strategic key performance indicators (KPIs). Rooted in the theories of Drucker [5] and Kaplan [6], these quantifiable metrics bridge high‐level strategy with daily operations, shifting the focus in R&D from monitoring outputs (e.g., number of experiments) to measuring outcomes (e.g., project progression).

With significant capital now flowing into AI‐driven technologies, such metrics have become essential for quantifying tangible value. In this context, KPIs must transcend technical validation—such as enrichment factors—to capture genuine pipeline impact [7]. Consequently, this perspective focuses on measuring how AI tools improve top‐line delivery in medicinal chemistry: identifying the right molecules effectively and shortening preclinical timelines. This approach aligns directly with Drucker's distinction between efficiency and effectiveness. As his famous adage notes, ‘There is nothing more wasteful than becoming highly efficient at doing the wrong thing.’ In drug discovery, prioritizing the selection of the right targets and leads—doing the right things—over simply optimizing processes for speed is key to impacting top‐line results [8].

While the principles of effectiveness remain constant, the methods for assessing them in computational chemistry have evolved over time. About a decade ago, several executives across different companies published papers discussing how the performance of computer‐aided drug design (CADD) scientists was assessed within their organizations. A key publication on the topic comes from researchers at Bristol Myers Squibb [9]. They describe a system of assigning project contributions of CADD scientists impact categories ranging from “data provided” to “enabling”. These are recorded in a database maintained by group leaders and fed by the CADD scientists themselves. Periodic cross‐evaluations help align a coherent view on impact. GSK used a similar approach [10] but not on an individual level. Key contributions by computational chemistry to decision‐making are recorded in an annual accomplishments report. The listed contributions are mainly those to compound designs in various phases of discovery and development as well as publications. Both Merck and Boehringer used more qualitative feedback by research team members to assess performance. At Merck, the question is “Did the design work lead to team success as expressed by the team members?” [11]. At Boehringer, team feedback was collected on the impact of the CADD member regularly to assess performance [12]. The outlined approaches from about a decade ago show that performance assessment was done on a more qualitative than quantitative level. Where categories of impact are assigned, their assignment is still a qualitative judgement of the assignee. Concepts have since been put forth to come to a more quantitative assessment: It has been suggested to bin KPIs for CADD scientists into process, quality, and communication [13]. For process, turn over time for repeated tasks or their efficiency can be monitored. This is especially applicable for routine tasks and can steer software and process development into a direction that frees up time for CADD scientists to focus on more innovative tasks. Quality measures can easily be derived from prospective evaluation such as using the model at the time of synthesis for predicting endpoints on a project level. Communication can be assessed by call outs from a defined user group (such as chemists), yet the impact remains unclear. One idea for assessing this is to use interviews or count mentions of the tool in design meetings. Beyond these aspects, efficiency or reduction of cost by CADD groups can be seen as the ante to get into the game. The real impact may come from creating innovative products [11], which is difficult to assess in the pharmaceutical industry, given the timelines for market success.

## 2 Drawbacks of KPIs

While KPIs are a very useful tool, especially for measurement and improvement of tasks with clearly defined processes and outcomes, application does not come without drawbacks [14]. Utilization of KPIs for performance evaluation makes them prone to Goodharts law [15]: “When a measure becomes a target, it ceases to be a good measure”. The organization may react in unanticipated ways to the mere act of measuring a certain KPI. Organizations with high goal alignment and high goal uncertainty such as pharmaceutical R&D are especially prone to unintended effects when KPIs are applied in a directive fashion for performance management [16]. Any formal measures of success require tracking and reporting, which may increase overhead. In addition, any KPIs must be well chosen, as too many may cause cherry‐picking and can cause detachment from the actual business, with the danger to move reality to what is on paper [17]. Misaligned incentives may be another drawback of KPIs creating conflicting interests through bounded rationality in an organization [18]. Utilization of KPIs for performance evaluation can also be detrimental to motivation, which is *sine qua non* for creativity [19]. The effectiveness of performance measurement is closely tied to the maturity of the field it is applied to. In emerging fields, greater flexibility is necessary to encourage exploration and allow for failure, as the criteria for success may still be undefined. As a field matures and transitions from product innovation to process innovation, established rules begin to take shape, and optimizing productivity becomes a priority [19]. The growing emphasis on KPIs in AI‐driven drug discovery—prompting us to write this perspective—suggests that the field is undergoing this shift. To foster creativity and innovation, it is crucial to avoid excessive measurement and micromanagement. However, for those seeking to maximize the existing benefits of these technologies, a stronger reliance on performance‐based evaluation could drive success.

## 3 Digital vs. Classic Projects

One common theme that emerged in our discussion was that ultimately, all companies desire an impact on high‐level efficiency KPIs such as time to clinics or cost (compounds, FTEs) per clinical asset. However, relying on these parameters to prove the value of digital tools is notoriously difficult, as they are composite measures influenced by countless confounding factors—from biological complexity to organizational friction.

To disentangle these factors and isolate the specific impact of AI, organizations often attempt to benchmark “digitally enabled” projects against “classic” projects (or historical baselines) or compare top‐line KPIs to “AI‐native” companies. However, this comparative approach faces a fundamental hurdle: standard KPIs typically flourish in environments where repeated tasks of comparable complexity are performed (e.g., manufacturing), whereas drug discovery is inherently idiosyncratic. Consequently, any comparison faces a significant signal‐to‐noise problem. There will always be substantial differences between projects—driven by target class, modality, and data availability—that complicate the analysis. Since the ideal “control project” (two identical teams working on the same target) will never exist, this runs the risk of conflating technological impact with target tractability. For instance, a “digital” project might succeed faster not because of the AI, but simply because the target was more druggable.

Despite these challenges, comparing “digital” versus “classical” approaches remains valuable if one can account for this variability. To move the field forward and enable fair comparisons, the EFMC^2^ encourages the development of a “Digitizability Score”—akin to druggability or ligandability ratings [20]. Such a score would act as a normalization factor, characterizing a target's amenability toward digital tools by weighing factors such as the availability of (co‐)structures, known ligands, and the precedence of the target class. Implementing such a score would allow organizations to prospectively select projects where digital leverage is highest. Furthermore, it would enable a more nuanced retrospective assessment, allowing management to distinguish between targets where AI could capitalize on rich data versus unprecedented targets where data scarcity limited impact. Ultimately, such a score allows KPIs to measure what matters: whether a digital tool delivered its maximum potential relative to the available data foundation—thereby restoring the utility of efficiency KPIs in a heterogeneous portfolio.

## 4 Adoption

As proposed a decade ago [13], many companies utilize usage metrics (e.g., model call‐outs, active user counts, workflow executions) to gauge adoption. While straightforward and real time, relying too heavily on usage as a proxy for impact is risky; as automation increases, human interaction decreases, rendering manual “usage” counts less meaningful to gauge the real impact of AI‐based tools. To drive and measure genuine adoption, we propose three impact‐focused KPIs.

**FIGURE 1:**
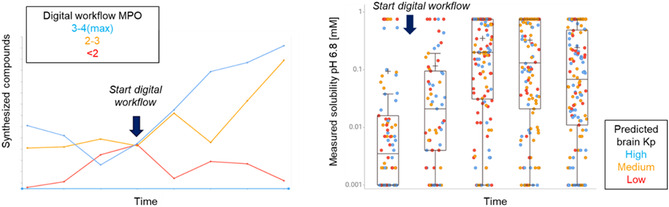
Representative plot showing an increase in compounds with desired MPO scores synthesized after introduction of a digital workflow (left). Increase in soluble compounds with desired brain Kp after introducing a digital tool (right).

A project‐level KPI widely applied in industrial medicinal chemistry is the tracking of key optimization parameters (KOPs)—such as potency or selectivity—over time (Figure 1). By plotting these values chronologically to determine the optimization velocity, one may identify a step‐change or discontinuity when a new digital tool was introduced to the project—a “Velocity Inflection.” A sharp improvement in the optimization velocity serves as objective proof that the tool (combined with the team's strategy) accelerated optimization. Feeding this data back to the team validates their effort and encourages carry‐over to future projects. Conversely, stagnation signals a need to pivot strategies.

An emerging trend observed by the group is replacing wet lab assays serving as stage gates in compound optimization with predictive models [21]. For example, in many test cascades across the industry, liver microsomes are used as a stage gate for the more expensive hepatocyte assay. If model performance is adequate in the project at hand, compounds with high confidence predictions of good microsomal stability can be directly measured in hepatocytes. The “Bypass Rate”—measured as the number or percentage of compounds successfully skipping a wet‐lab assay based on a prediction—serves as a dual metric. It quantifies both efficiency (time/cost saved) and trust (the team's willingness to bet resources on a prediction).

Finally, adoption is fundamentally about user experience. The Net Promoter Score (NPS), a standard metric in customer experience management [22], can be adapted for internal R&D tools. By asking project teams, “On a scale from 0 to 10, how likely are you to recommend this tool to a colleague?” organizations can generate an “Internal NPS” (iNPS). Since project teams are the “customers” of CADD, a high iNPS is a leading indicator of future adoption and spread of tools within the organization, making it a worthwhile KPI to optimize. In addition, one could profit from the wealth of experience with this KPI in the service industry [23].

## 5 Assessing Impact: From Technical Metrics to Strategic KPIs

To effectively evaluate the value of modern AI‐based tools, it is necessary to distinguish between technical evaluation metrics (which assess model quality) and strategic KPIs (which assess business impact). This distinction is particularly critical because the transition from classical CADD to modern AI has shifted the nature of the work.

Classical CADD methods—such as docking or pharmacophore modeling—are primarily expert‐driven tools; their success was linked to the manual inputs and hypothesis generation of the modeler. In this context, “expert impact” (qualitative feedback) was often the most appropriate metric. In contrast, modern AI technologies operate as advanced decision support systems. They do not merely evaluate human hypotheses; they proactively generate new molecular ideas and prioritize experimental designs for review. This shift from passive tool to active “augmented intelligence” creates a new requirement for system‐level KPIs that measure the utility of these automated suggestions—metrics that traditional validation scores cannot capture.

While strategic KPIs measure value, assessing the technical quality of predictive models remains a necessary prerequisite. For regression tasks (common in ADME/property prediction), metrics such as R^2^ and mean absolute error are standard. For classification tasks, Matthews Correlation Coefficient (MCC) and Cohen's kappa provide robust measures of accuracy. However, static test sets often fail to capture real‐world utility. To align these metrics with project reality, it is advisable to evaluate them on a time‐split basis, using the exact model version available at the time of synthesis to simulate prospective performance. While high scores on these metrics are necessary, they are insufficient as KPIs because they do not measure whether the model actually improved the decision‐making of the research team. The following sections detail specific strategic KPIs designed to measure this new layer of impact across different digital tools.

## 6 Virtual Screening

For virtual screening, technical metrics like enrichment factors are insufficient because they do not capture the value of the chemical matter found. A more robust strategic KPI is the per‐target comparison of success rates against experimental high‐throughput screening (HTS), which is more time‐consuming and costly. To enable this, VS campaigns should be run in parallel with wet‐lab screens wherever feasible to correct for target bias and an adequate amount of virtual hits must be acquired and tested. When screening large virtual chemical spaces, several hundred compounds to be acquired/synthesized were recommended by the group. When virtually screening internal compound libraries, assay throughput is the only restricting factor, and the number can be increased substantially. A critical KPI to define success is the “Team Uptake Rate”—the percentage of hits that are actually selected by medicinal chemists for further optimization. Hits that are technically active but structurally unattractive (and thus ignored by the team) add no value. While uptake is a composite measure (influenced by perceived quality and timing), it remains a useful filter for value.

In addition to screening time and success rates, cost per hit cluster could serve as another metric for comparing screening technologies. While *in silico* methods may initially seem more cost‐efficient, they can incur additional expenses such as software licensing fees and the complex synthesis of possibly false‐positive hits. Utilization of such a metric as KPI would encourage cost‐effective utilization of screening technologies.

## 7 GenAI

Unlike predictive models, generative AI lacks a ground truth to measure against. Therefore, technical metrics are often replaced by proxy metrics for quality (e.g., validity, synthesizability scores). However, we put forth “Attributed Impact,” as a strategic KPI for GenAI. This is defined as the number of AI‐proposed compounds (or fragments) that are subsequently synthesized and advanced by research teams. For example, once a compound idea or fragment can be confidently linked to GenAI's suggestion, tracking how often it appears incorporated in synthesized compounds provides a valuable metric for judging the impact of the system. But in practice, using such metrics may be complicated due to the size of chemical space and often small overlap between both design spaces [24]. Teams often refine AI suggestions, obscuring the link between the tool and the final molecule. A strict “exact match” KPI underestimates impact, while a loose metric overestimates it (claiming credit for obvious ideas). A balanced KPI could track “Novelty‐Weighted Uptake.” This measures synthesized compounds that (a) originated from GenAI and (b) demonstrate a defined dissimilarity threshold (e.g., Tanimoto < 0.8) from the project's existing chemical space. This ensures the KPI rewards innovative suggestions rather than just identifying obvious analogs and reduces the likelihood that the team would arrive at the same sufficiently dissimilar compound idea as the tool on their own.

## 8 ADME Models

For predictive ADME models, the goal is not just accurate prediction but decision support. The fundamental KPI is the reduction in the synthesis of compounds with undesirable profiles. This can be monitored by tracking the “Mean Property Shift,” the moving average of key endpoints (e.g., metabolic stability, permeability) of synthesized compounds over time. If the model is impactful, this average should trend towards desirable values. A lack of improvement in this KPI indicates essentially one of two failures: low model quality (technical failure) or low adoption (cultural failure). If technical metrics such as time‐split R^2^/MCC are high but the “Mean Property Shift” is flat, the focus must shift to KPIs in the area of adoption to ensure the models are actually being used to filter designs. Ensuring correct prospective deployment is critical, as high‐quality models may nonetheless underperform if not utilized effectively. An equally important consideration is the maintenance of core operational KPIs, including service uptime, robustness of the predictive framework, and response times, as these parameters are essential for ensuring consistent, real‐time utilization of the predictive tools.

## 9 Outlook

We recognize that our industry is at a transformational juncture, which may prompt us to rethink traditional KPIs in light of AI's vast potential. In the future, rather than relying solely on classical measures of progress (like optimization of multiparameter scores or key optimization parameters), we might gauge project success also by how rapidly model performance improves. This forward‐thinking approach encourages a steep learning curve right from a project's outset and fosters parabolic rather than linear advancements, potentially saving time and reducing costs over the long haul.

To develop KPIs towards more reliable predictors of success, longitudinal correlation studies could investigate the link of early‐stage strategic KPIs (such as “Velocity Inflection” or “Bypass Rate”) to top‐line deliverables such as efficiency and speed. Further, defining and standardizing the proposed “Digitizability Score” by determining which data factors (e.g., structural availability vs. assay variance) best predict AI amenability would enable adequate benchmarking. Finally, exploring the human–AI interaction—specifically how behavioral aspects like trust influence decision quality—would provide insights for augmented intelligence systems.

Moreover, our current ability to use pre‐AI projects as a baseline uniquely positions us to assess AI's impact—a measurement opportunity that will fade as these projects recede into history.

## 10 Conclusion

Although KPIs are often viewed with skepticism by scientists [9], they remain a fundamental management tool. We believe that, when proactively and thoughtfully designed, KPIs can play a useful role in tracking the impact of predictive tools, improving their adoption and fostering innovation in pharmaceutical R&D. By providing quantitative insights, KPIs can help decision‐makers assess the value of predictive approaches, ultimately accelerating the development of innovative medicines and reducing cost. Table 1 provides a summary of the KPIs put forth in this perspective, which are designed to capture the distinct value drivers of modern digital tools.

**TABLE 1:** Proposed Strategic KPIs for AI in Lead Optimization.

Area	KPI Name	Definition / Measurement Approach	Strategic Goal
Virtual screening	Team uptake rate	Percentage of AI‐proposed hits selected by chemists for synthesis or further testing	Measure the perceived value and relevance of virtual hits beyond theoretical enrichment
Virtual screening	Cost per validated Hit	Total screening cost (compute + synthesis) divided by the number of biologically confirmed hits or clusters	Ensure AI screening is cost‐effective compared to traditional methods
Generative AI	Novelty‐weighted impact	Count of synthesized compounds that (a) originated from GenAI and (b) meet a novelty threshold (e.g., Tanimoto < 0.8 vs. known compounds)	Quantify the system's contribution to novel chemical matter (innovation) vs. rote exploitation
ADME/property prediction	Mean property shift	Moving average of measured endpoints (e.g., metabolic stability) for synthesized compounds over time	Assess if predictive models are effectively driving the project or the departmental collective of synthesized compounds toward desired property profiles
Adoption	Velocity inflection	Step‐change in the slope of key optimization parameters (KOPs) plotted over project time	Objectively measure if a tool accelerated the rate of lead optimization
Adoption	Bypass rate	Percentage of compounds skipping a wet‐lab stage‐gate assay based on high‐confidence prediction	Measure efficiency gains and team trust in predictive reliability
Adoption	Internal NPS (iNPS)	”How likely are you to recommend this tool?” (0–10 Scale)	Indicator of user satisfaction and future organizational adoption

As researchers in the pharmaceutical industry, we recognize that even defined metrics like those above are not perfect tools and may sometimes oversimplify complex scientific processes. However, when aligned with the goals of science and innovation, they can serve as a bridge between research efforts and corporate decision‐making. While not without their limitations, KPIs can be a helpful tool to improve the adoption of predictive models and inform teams on the success of the optimization strategy they apply. Thereby, they support the development of innovative medicines that reach patients faster, more affordably, and—ideally—with greater reliability and consistency.

This raises a broader question: given the inherent complexity and variability of biological systems, can we ever achieve the same level of predictability and control in biopharmaceutical research as we do in software engineering, digital technologies, or other industries? Venture capital flows indicate a strong belief in this possibility—hoping that computational advances will make drug discovery more systematic and scalable. Pushing the boundaries to which extent this is possible and thus can be captured in KPIs remains an open and fascinating challenge.

## Conflicts of interest

All authors are employees of companies applying AI‐driven workflows in drug discovery and apply these in their work. M. Krier is an employee of Cadence Molecular Sciences, a software vendor for AI‐based solutions for drug discovery.

## Disclaimer

The opinions expressed in this publication are the view of the author(s) and do not necessarily reflect the opinions or views of *ChemMedChem*, the Publisher, Chemistry Europe, or the affiliated editors.
